# Bullous cutaneous reaction mimicking Stevens-Johnson syndrome due to Nigella sativa supplement ingestion

**DOI:** 10.1016/j.jdcr.2026.06.009

**Published:** 2026-06-18

**Authors:** Brian Chu, Neha Kinariwalla, Christopher P. Elco, Su-Jean Seo

**Affiliations:** a Department of Dermatology, Warren Alpert Medical School, Brown University, Providence, Rhode Island; b Department of Pathology and Laboratory Medicine, Warren Alpert Medical School, Brown University, Providence, Rhode Island

**Keywords:** adverse reaction, black cumin seed oil, bullous dermatosis, cutaneous drug reaction, dietary supplements, herbal supplements, Nigella sativa, Stevens-Johnson syndrome

## Introduction

Despite federal rules barring manufacturers from claiming that supplements can diagnose, cure, treat, or prevent disease, many patients increasingly incorporate them into daily regimens. While most supplements are well-tolerated, several have notable dermatological adverse effects. One example is black cumin seed oil, or *Nigella sativa* oil (NSO). Topical NSO has been reported to cause severe cutaneous reactions mimicking Stevens-Johnson syndrome/toxic epidermal necrolysis (SJS/TEN). Here, we report a case of ingestion of a supplement containing NSO leading to a bullous cutaneous reaction with features overlapping with SJS.

## Case description

A 69-year-old man with type 2 diabetes, chronic kidney disease, and Parkinson disease presented with 5 days of rapidly progressive rash, beginning on the chest and face and spreading to the trunk and mucosal surfaces. He described stinging discomfort like sunburn but denied frank skin pain. He was directed to the emergency department by an outpatient dermatologist, who was concerned for SJS/TEN.

He reported starting a supplement purchased online 10 days prior, purportedly to improve his immune system. The ingredient list included oil of oregano (*Origanum minutiforum*), black seed oil (*N sativa*), carvacrol (a phenol present in oil of oregano), and thymoquinone (a quinone present in black seed oil). He otherwise denied any new medications or exposures. His chronic home medications included carbidopa-levodopa, citalopram, metformin, olmesartan, omeprazole, rosuvastatin, finasteride, tamsulosin, and vibegron.

**Fig 1:**
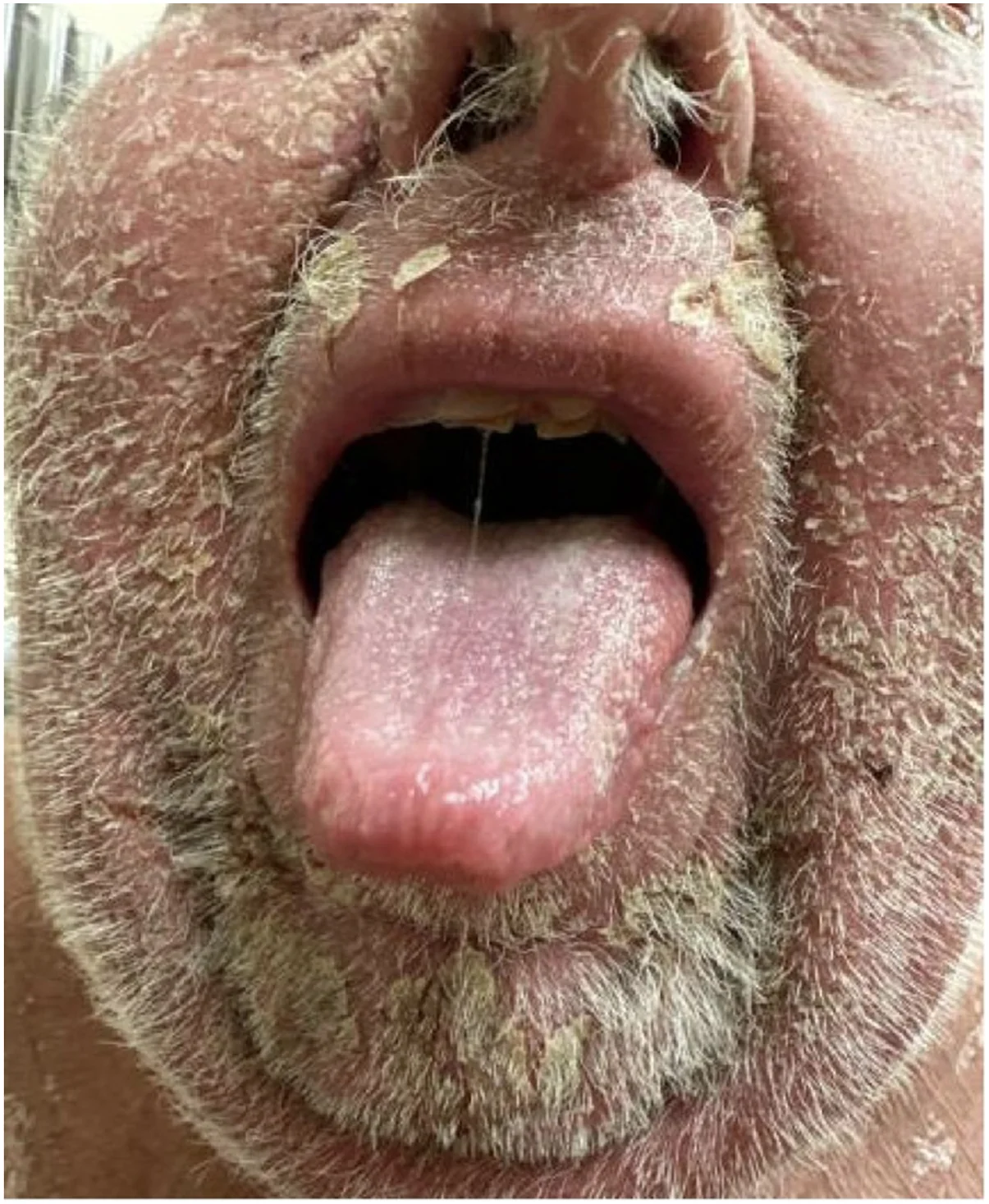
Diffuse scaling and erythema on the face without mucosal involvement.

**Fig 2:**
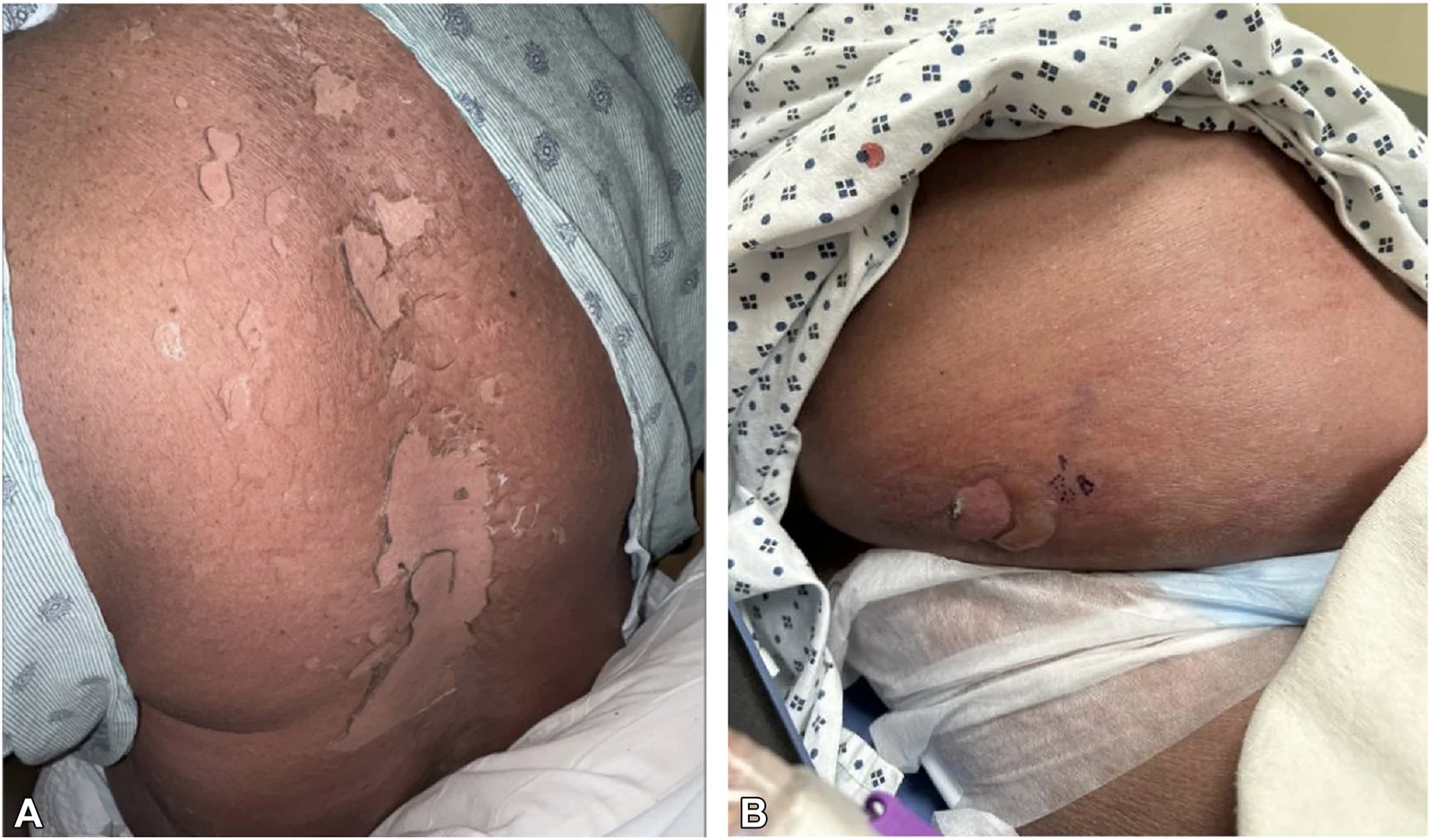
Flaccid, full thickness subepidermal bullae on the **(A)** back and **(B)** lower abdomen.

**Fig 3:**
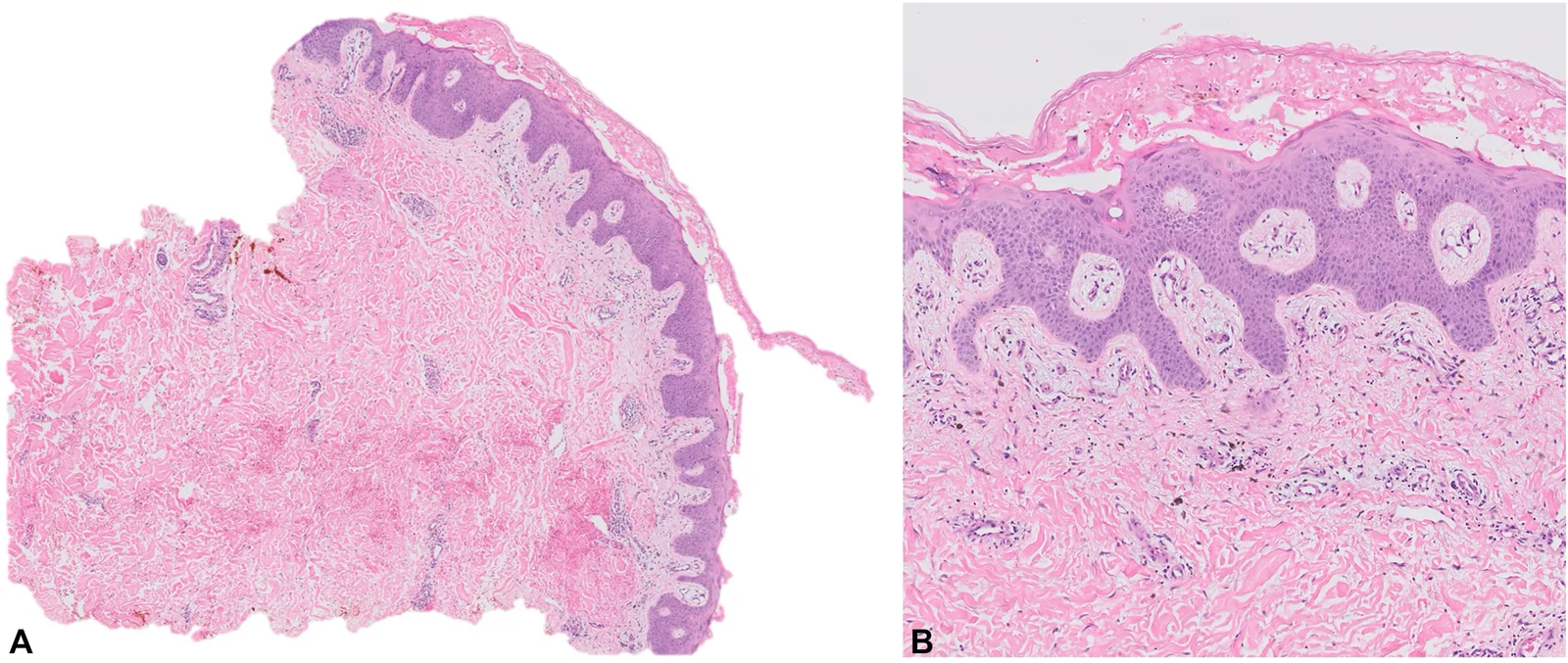
Histopathological examination demonstrating full-thickness epidermal necrosis overlying re-epithelialized epidermis. Sparse perivascular infiltrate with scattered eosinophils was present.

Examination revealed diffuse scaling and erythema on the face and neck (Fig 1). Chronic ectropion was noted. Flaccid full-thickness subepidermal bullae were noted on the lower abdomen, popliteal fossae, glans penis, and perianal skin (Fig 2). His oral mucosa was unaffected. A skin biopsy of an abdominal bulla demonstrated near full-thickness epidermal necrosis overlying re-epithelialized epidermis (Fig 3). Sparse perivascular infiltrate with scattered eosinophils was present. A resolving bullous vacuolar interface dermatitis was suggested.

Given the initial clinical concern for SJS, a potentially life-threatening diagnosis, he empirically received 1 dose of etanercept before a definitive diagnosis was reached. However, the absence of significant skin pain and dusky lesions, reassuring laboratory studies and vitals, lack of a classic culprit medication that would cause SJS, and overall well appearance raised doubt. Upon review of the literature of cutaneous reactions to NSO, a diagnosis of NSO-induced bullous cutaneous reaction was made. He was treated with topical triamcinolone ointment wet wraps. He was not treated with any other systemic medications other than intravenous fluids and his home medications. He improved rapidly and was discharged 3 days later, which would be unusual for SJS.

He was referred to our contact dermatitis clinic due to concern for systemic contact dermatitis from NSO ingestion as the underlying mechanism of the bullous cutaneous reaction, but he had discarded the culprit capsules after the hospitalization and did not wish to risk another severe rash with patch testing. The available commercial assays did not encompass the culprit ingredients.

## Discussion

*N sativa* oil (NSO) is a widely used herbal supplement with claimed benefits for hypertension, asthma, diabetes, and arthritis.^1^ Several case reports have described severe bullous cutaneous reactions to *N sativa* (Table I). One case described a patient hospitalized for a vesiculobullous rash after 2 weeks of topical application and ingestion of *N sativa* oil.^2^ Patch testing for the oil was strongly positive, and the patient was diagnosed with systemic and contact bullous drug eruption. Another case described topical application of *N sativa* resulting in an SJS-like presentation with widespread desquamation.^5^ A case series of 3 patients described similar presentations after topical *N sativa* oil exposure, specifically the thymoquinone component.^3^ One unusual case reported SJS/TEN overlap with bullous pemphigoid secondary to *N sativa* oil ingestion.^4^ No similar reports exist for *Origanum minutiflorum*, hence why we suspect NSO as the culprit.

**Table I:** Multiple case reports and series have demonstrated a range of severe cutaneous reactions to topical application or ingestion of Nigella sativa-containing substances

Case	Patient	Route of exposure	Diagnosis	Histology	Treatment
[This case]	69 M	Oral ingestion	Bullous cutaneous reaction	Near full thickness epidermal necrosis overlying re-epithelialized epidermis. Sparse perivascular infiltrate with scattered eosinophils.	Topical steroids wet wraps
Gelot et al 2012^2^	53 F	Oral ingestion and topical application	Systemic and contact bullous drug eruption	Subepidermal detachment and necrosis of the epidermal roof consistent with lesions of the EM-TEN spectrum.	Topical clobetasol
Gaudin et al 2018^3^	40s F	Topical application	Severe acute contact dermatitis	Epidermal apoptosis with vacuolar alteration of the basal layer, keratinocyte apoptosis, and moderate perivascular infiltrate of lymphocytes in the dermis	Topical clobetasol and petrolatum
Gaudin et al 2018^3^	20s F	Topical application	Severe acute contact dermatitis	Epidermal apoptosis with vacuolar alteration of the basal layer, keratinocyte apoptosis, and a moderate perivascular infiltrate of lymphocytes in the dermis	Topical clobetasol and petrolatum
Gaudin et al 2018^3^	20s F	Topical application	Severe acute contact dermatitis	Epidermal apoptosis with vacuolar alteration of the basal layer, keratinocyte apoptosis, and a moderate perivascular infiltrate of lymphocytes in the dermis	Topical clobetasol and petrolatum
Bonhomme et al 2016^4^	54 F	Oral ingestion	SJS/TEN, and subsequent bullous pemphigoid	Biopsy 1: epidermal necrosis, subepidermal blister, abundant melanophages, and negative direct immunofluorescence Biopsy 2: subepidermal blister with eosinophils, linear C3/IgG at dermoepidermal junction	Topical clobetasol

In our case, we believe the patient’s incontinence and diaper use due to Parkinson disease contributed to the distribution of the lesions. The excretion of thymoquinone metabolites in the urine or stool may explain the presence of bullae primarily on the lower body, and particularly the anogenital skin.^6^ We could not perform confirmatory patch testing due to patient preference. Therefore, a definitive diagnosis of systemic contact dermatitis could not be reached in this case.

Limited regulatory oversight, particularly online products, increases the risk of unrecognized exposures. A 2015 study estimated that 23,000 emergency department visits annually in the US were linked to dietary supplements.^7^ Although the Food and Drug Administration (FDA) does not require supplement companies to prove safety or efficacy before bringing products to market, we encourage the FDA to consider issuing a safety alert for cutaneous reactions to *N sativa* products, now that there is a clear set of cases in the literature. Dermatologists should continue to include over-the-counter and online supplements in history taking when suspecting a drug-induced rash, not only prescription medications.

In summary, we present a case of ingestion of Nigella sativa supplements resulting in a bullous cutaneous reaction with features overlapping with SJS.

## Conflicts of interest

None disclosed.
